# The Truncated Isoform of Somatostatin Receptor5 (sst5TMD4) Is Associated with Poorly Differentiated Thyroid Cancer

**DOI:** 10.1371/journal.pone.0085527

**Published:** 2014-01-21

**Authors:** Manel Puig-Domingo, Raúl M. Luque, Jordi L. Reverter, Laura M. López-Sánchez, Manuel D. Gahete, Michael D. Culler, Gonzalo Díaz-Soto, Francisco Lomeña, Mattia Squarcia, José Luis Mate, Mireia Mora, Laureano Fernández-Cruz, Oscar Vidal, Antonio Alastrué, Jose Balibrea, Irene Halperin, Dídac Mauricio, Justo P. Castaño

**Affiliations:** 1 Service of Endocrinology and Nutrition, Department of Medicine, Germans Trias i Pujol Health Science Research Institute and Hospital, Universitat Autònoma de Barcelona, Badalona, Spain; 2 Department of Cell Biology, Physiology and Immunology University of Córdoba, Reina Sofía University Hospital, Instituto Maimónides de Investigación Biomédica de Córdoba (IMIBIC), CIBER Fisiopatología de la Obesidad y Nutrición (CIBERobn), Córdoba, Spain; 3 IPSEN, Milford, Massachusetts, United States of America; 4 Service of Endocrinology, Hospital Clínico de Valladolid, Valladolid, IEN-UVa, Valladolid, Spain; 5 Service of Nuclear Medicine, Hospital Clínic de Barcelona, Barcelona, Spain; 6 Service of Radiology, Hospital Clínic de Barcelona, Barcelona, Spain; 7 Department of Pathology, Germans Trias i Pujol Health Science Research Institute and Hospital, Universitat Autònoma de Barcelona, Badalona, Spain; 8 Service of Endocrinology and Nutrition, Hospital Clínic de Barcelona, Barcelona, Spain; 9 Service of Surgery, Hospital Clínic de Barcelona, Barcelona, Spain; 10 Service of General Surgery, Department of Surgery, Germans Trias i Pujol Health Science Research Institute and Hospital, Universitat Autònoma de Barcelona, Badalona, Spain; 11 Service of General Surgery, Department of Surgery, Vall d'Hebron Research Institute and Hospital, Universitat Autònoma de Barcelona, Barcelona, Spain; University of Santiago de Compostela School of Medicine - CIMUS, Spain

## Abstract

Somatostatin receptors (ssts) are expressed in thyroid cancer cells, but their biological significance is not well understood. The aim of this study was to assess ssts in well differentiated (WDTC) and poorly differentiated thyroid cancer (PDTC) by means of imaging and molecular tools and its relationship with the efficacy of somatostatin analog treatment. Thirty-nine cases of thyroid carcinoma were evaluated (20 PDTC and 19 WDTC). Depreotide scintigraphy and mRNA levels of sst-subtypes, including the truncated variant sst5TMD4, were carried out. Depreotide scans were positive in the recurrent tumor in the neck in 6 of 11 (54%) PDTC, and in those with lung metastases in 5/11 cases (45.4%); sst5TMD4 was present in 18/20 (90%) of PDTC, being the most densely expressed sst-subtype, with a 20-fold increase in relation to sst2. In WDTC, sst2 was the most represented, while sst5TMD4 was not found; sst2 was significantly increased in PDTC in comparison to WDTC. Five depreotide positive PDTC received octreotide for 3–6 months in a pilot study with no changes in the size of the lesions in 3 of them, and a significant increase in the pulmonary and cervical lesions in the other 2. All PDTC patients treated with octreotide showed high expression of sst5TMD4. ROC curve analysis demonstrated that only sst5TMD4 discriminates between PDTC and WDTC. We conclude that sst5TMD4 is overexpressed in PDTC and may be involved in the lack of response to somatostatin analogue treatment.

## Introduction

Thyroid cancer in its papillary and follicular well differentiated forms (WDTC) have an excellent prognosis with very high cure rates after surgical treatment followed by radioiodine [1], [2]. However, the management of patients with poorly differentiated (PDTC) radioiodine (RAI)-refractory thyroid cancer is controversial and difficult, with no effective treatment available until the recent appearance of multi-target tyrosine kinase inhibitors [3]. It is usually accepted that PDTC occurs either *de novo* or progress from a pre-existing WDTC through a multistep process of genetic and epigenetic changes. New molecular pathways and targets are under research to discover the dedifferentiation process of thyroid cancer cells [4].

Somatostatin is a ubiquitous peptide from the central nervous system-gastrointestinal tract that exerts many biological functions in both endocrine and non-endocrine tissues by binding to its five receptor subtypes (sst1–5) [5]. Expression of somatostatin receptors (ssts) in tumors arising from different tissues has been the basis of using somatostatin analogues as a bio-therapeutic option thereby leading to stabilization of tumor growth and increasing patient survival time in some types of cancer [6], [7]. Recent and old studies indicate the presence of ssts in samples of different pathological conditions of the thyroid gland including non-medullary thyroid cancer [8], [9]

The sst receptor family has been fully characterized, and classically, 5 subtypes have been identified with different biological implications in cell cycle [8]. Sst subtypes 2 and 5 are involved in the control of hormone secretion as well as cell growth, while sst1, 3 and 4 are mostly implicated in non-hormonal functions [10]–[12]. Somatostatin analogue treatments used in clinical practice involve binding of sst2 and 5 with different affinity [13], [14], with sst2 having the maximal binding affinity to the most currently used compounds, octreotide and lanreotide [15], while pasireotide is a new compound targeting sst5 with higher affinity [16], [17]. Recently, two new functional human sst5 variants have been identified, the so-called sst5TMD4 and sst5TMD5, generated through non-canonical splicing, and displaying unique molecular/functional features [18], [19]. Interestingly, sst5TMD4 has been detected in patients with GH-secreting adenomas and breast cancer [19]–[21]. sst5TMD4 expression has been linked to poor response to octreotide treatment in acromegaly. However, its functional significance is not well described in other kind of tumors.

Here, we conducted a retrospective study at the molecular level to explore the potential association of sst subtypes phenotype, and particularly sst5TMD4, in WDTC and PDTC patients, its potential use as a biological marker and its therapeutic implications.

## Materials and Methods

The study was performed according to the principles of the Declaration of Helsinki, (revision of Edinburgh 2000) and was approved by the Human Research Ethics Committee of the Hospital Clinic de Barcelona (Comité Ético de Investigación Clínica, CEIC), Barcelona, Spain. The nature of the study was explained to the patients, and written informed consent was obtained in each case.

### Patients

The study group consisted in 39 patients (29 women/10 men) from two university hospitals. Among these 39 subjects, 20 patients had PDTC (table 1) with mean age 57±13 years, (12 women/8 men); from those 20 PDTC cases, 18 were papillary tumors and 2 were of follicular in origin. All had active disease (stage IV of TNM classification), with a follow-up ranging from 6 to 21 years and 9 of them died during follow up. Thyroglobulin concentrations in these patients ranged from 4 to 29595 ng/ml. All patients were treated with a similar therapeutic protocol following international guideline recommendations [1], [2]. Those patients bearing PDTC required multiple surgeries when indicated and more than one dose of RAI; RAI therapy was no longer used when RAI uptake became negative despite disease progression. PDTC patients were treated with thyroxine at suppressive doses while well differentiated cases received thyroxine at a dose targeting TSH a normal-low value (<2 mIU/ml). All PDTC cases showed negative RAI trapping capacity during their evolution and all had locoregional neck recurrence and/or extracervical metastases, either in the central nervous system, bone, lung or mediastinum detected by different imaging procedures including ultrasonography, CT-scan, MRI, and ^18^F-FDG-PET scan. In order to participate in a pilot study in which somatostatin analogue octreotide would be administered, in 11 out of these 20 patients, a ^99^mTc-depreotide scintigraphy was performed for evaluation of sst presence in the residual primary tumor tissue and metastases. Five out of the 11 patients in which the ^99^mTc-depreotide scintigraphy showed a positive image of the residual primary tumor and/or metastatic tissue and progressive disease by RECIST criteria received treatment with the long acting somatostatin analogue octreotide at doses of 30 mg IM/month for a period of 3–6 months during 2007 and 2008 (Table 1).

**Table 1:** Demographic and laboratorial data and copy number of ssts subtypes (sst1–5 and truncated sst5TMD5 and sst5TMD4 variants) mRNA content in poorly differentiated thyroid cancer cases.

			Absolute mRNA copy number/50 ng of total RNA									
PDCT	S	A	sst1	sst2	sst3	sst4	sst5	sst5TMD5	sst5TMD4	Thyroglobulin (ng/mL)	Metastases location	Depreotide
1*	F	46	81	229	0	20	20	0	1200	180	neck, lung	lung+,neck+
2*	M	76	95	38	69	40	23	0	99	2220	lung, bone	lung−
3	F	44	135	7	13	31	30	0	5154	4	neck, lung	lung+, neck+
4	F	56	42	9	5	20	14	0	581	28	neck, lung	lung+, neck+
5	M	55	90	103	93	31	18	0	139	34	neck, lung	lung−, neck+
6*	M	48	0	155	40	59	33	0	1410	73	neck, lung, bone	lung−, neck+
7	F	35	86	33	23	42	13	0	810	174	neck, lung	lung+, neck+
8*	F	58	134	88	93	41	22	0	192	588	neck, lung	lung−, neck+
9	M	66	74	45	41	27	10	0	27	33	lung, bone	lung−,neck−
10	M	35	141	43	72	32	31	0	1489	18	neck, lung	lung+, neck+
11*	F	57	275	249	509	170	41	0	666	290	neck, lung	lung−, neck+
12*	F	68	174	141	166	60	69	0	728	430	lung, bone	NP
13	M	69	156	250	79	63	12	0	1	18	neck, lung	NP
14	F	54	5	4	0	2	0	0	2	25	neck, lung, bone	NP
15	F	58	53	140	0	11	19	0	3384	35	neck	NP
16	F	42	62	54	19	28	10	0	38	89	neck	NP
17*	F	59	196	164	40	68	51	0	303	29595	neck lung, bone	NP
18	M	65	270	745	191	12	12	0	10659	1450	neck, lung	NP
19*	F	68	212	110	24	78	49	0	387	250	neck	NP
20*	M	66	0	547	0	0	0	0	35957	389	neck, lung, bone	NP
**Median**	**56**	**114**	**158**	**74**	**42**	**24**	**0**	**3661**				

PDCT: poorly differentiated thyroid cancer; S: Sex; A: Age; All PDCT were papillary tumors except number 11 and 15 which were of follicular in origin. All patients had active disease (stage IV of TNM classification).

* Patients that died during follow-up.

The remaining 19 of the 39 patients included in the study constituted the control group; all had well differentiated tumors, being free of disease at the time of the study and apparently cured after the first and single treatment performed at the time of diagnosis, consisting in total thyroidectomy followed by 100 mCi of RAI given 3 months after surgery. Mean age of this group was 48±8 years (10 men and 10 women).

### Imaging techniques

^99^mTc-depreotide scintigrams were performed in 11 out 20 patients bearing a PDTC (table 1). Depreotide is a somatostatin analogue binding to SSTR 2, 3, and 5 on cell surfaces, more efficiently than octreotide radiolabelled compounds. All patients were on hormonal suppressive therapy with thyroxine when the imaging procedures were performed. ^99^mTc-depreotide was prepared from a commercially available kit (NeoSpect®; Amersham Health, Buckinghamshire, UK). The iv-injected dose per patient was 740 MBq (50 µg peptide) of ^99^mTc-depreotide. Scintigraphic imaging was acquired with a double-head gamma camera and a high-resolution collimator. ^99^mTc-depreotide images were obtained at 1 h and 3 h after injection. Planar acquisitions of total body and single-photon emission computer tomography (SPECT) of head, neck, thorax, and abdomen were performed. In order to evaluate hypermetabolic tumor activity, ^18^F-FDG-PET scans were additionally obtained at 1 h after iv injection of 370 MBq 18F-FDG using a dedicated full-ring PET scanner, with an axial field of view of 15.2 cm and a maximum resolution of 3.8 mm transaxially and 4.0 mm axially.

### Expression of ssts in thyroid cancer tissue and normal thyroid gland

Determination of ssts expression by quantitative real-time RT-PCR (qrtRT-PCR) was performed in tumor paraffin-embedded samples of all WDTC and PDTC patients. In PDTC patients, samples were obtained at the time of surgical reintervention for advancing disease state from tumoral tissue of cervical recurrence in all cases. Additionally, samples of normal thyroid gland (n = 7) obtained from the Tumor Biobank of the Germans Trias Hospital and from a pool of samples of normal thyroid gland obtained at the University of Córdoba were also included in the study. Specifically, tumor and normal thyroid samples (4 sections of 5 µm thick) were processed for a recovery of total RNA using the RNeasy FFPE Kit, with DNAase treatment (Qiagen, Barcelona, Spain). Details of RNA quantification, reverse transcription (RT; 1 µg of total RNA) and qrtRT-PCR for all sst-subtypes have been previously reported in detail 18,22. It should be noted that a specific standard curve for each transcript was run with each set of samples to estimate copy number. Total RNA samples that were not reverse-transcribed were run to control for genomic and/or technical DNA contamination (background). To control for variations in the amount of RNA used in the RT reaction and the efficiency of the RT reaction, the expression level (copy number) of 3 housekeeping genes (glyceraldehyde-3-phosphate dehydrogenase – GAPDH, β-actin and 18S) was determined for each sample as previously reported (19,20). GeNorm® 3.3 visual basic application for Microsoft Excel® (http://medgen.ugent.be/jvdesomp/genorm) [23] was used to assess the stability of the housekeeping genes. The geometric means of the copy numbers for GAPDH and β-actin within each sample were used as a final normalization factor (NF). Results were then reported as sst copy number minus background divided by the corresponding NF.

### Response to octreotide treatment

A subgroup of PDTP patients refractory to radioiodine treatment, progressive disease by radiological criteria and positive depreotide scans received octreotide treatment for 3–6 months in a pilot compassionate protocol accepted by the patients and the hospital ethics committee (Table 1). Efficacy was evaluated by means of the RECIST criteria (http://www.recist.com/index.html) and according to ssts subtypes.

### Statistical analysis and Receiver Operating Characteristic (ROC) curves of ssts expression

Categorical data were described using frequencies and percentages. For quantitative variables, measures of central tendency and dispersion (mean and standard deviation) are provided.

Bivariate analysis of the main variables was performed using 2×2 contingency tables with χ2 tests to evaluate significant differences in frequencies for categorical variables. The parametric t-test was used to compare quantitative data. In those quantitative variables not normally distributed and the non-parametric Mann Whitney's U test was used when indicated.

All analyses were carried out using the Statistical Package for Social Sciences (SPSS) version 17.0 (SPSS®, Chicago, IL, USA). We considered p<0.05 to be statistically significant.

ROC (Receiver Operating Characteristic) was performed for evaluation of diagnostic test sensibility and specificity. It was used as a tool to measure how well the expression of ssts could distinguish between the two diagnostic groups (patients with active disease/patients free of disease). The statistical analysis of the ROC curves was performed by calculating the Area Under the Curve (AUC) of each receptor, and comparing them with the AUC of the reference line using student-t test.

## Results

### Ssts identification in poorly differentiated thyroid cancer by ^99^mTc-depreotide imaging

**Figure 1:**
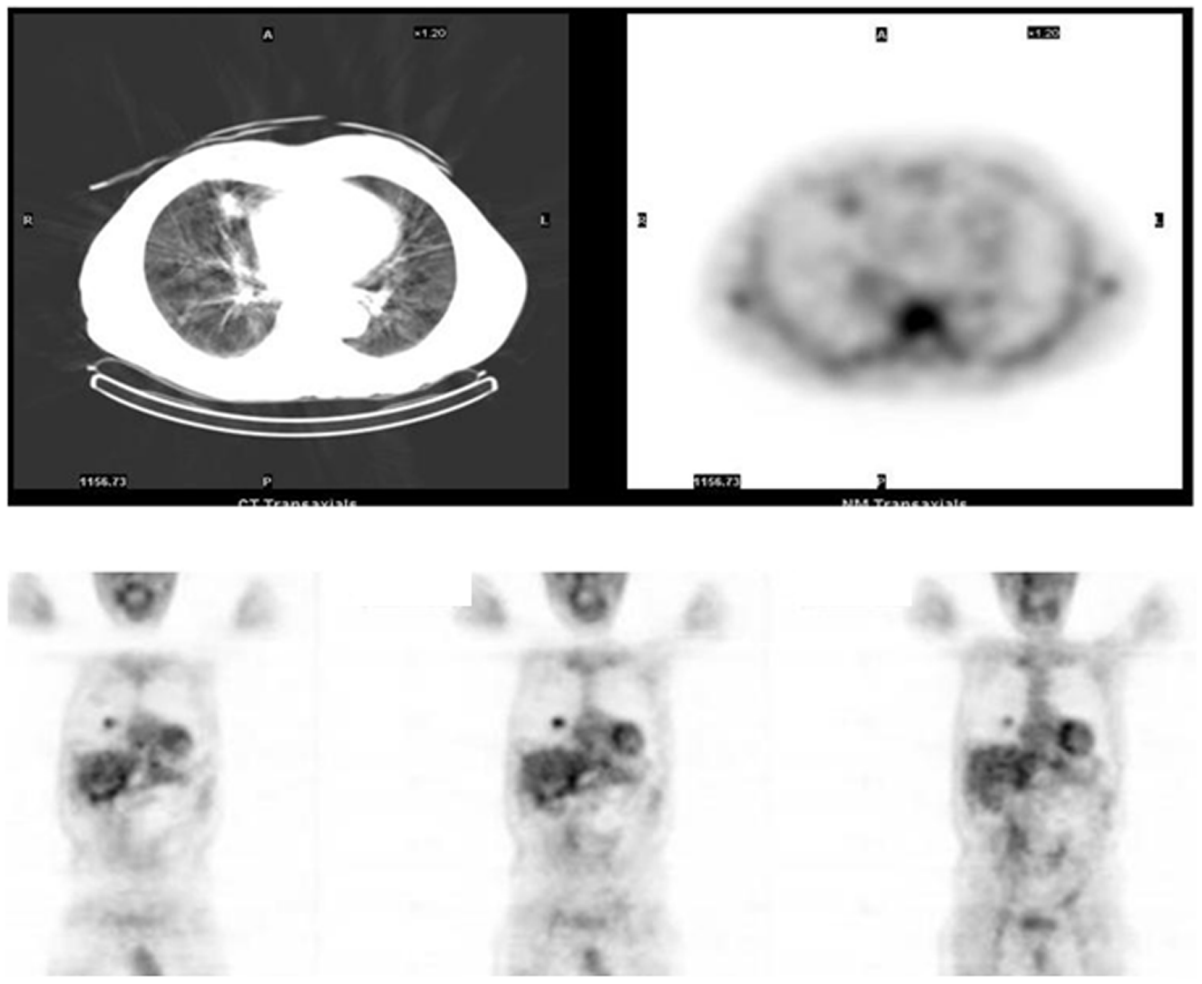
99mTc-depreotide scintigraphy (patient 10 in table 1) showing positive images of lung metastases.

In the 11 patients (5 men and 6 women) from the PDTC group in which a ^99^mTc-depreotide was performed, 9 showed cervical loco-regional recurrence and all presented metastases of difference size in the lungs or bone identified either by CT scan and/or MRI and/or bone scintigraphy. ^99m^Tc-depreotide scintigraphy was positive in the neck in 6 out of these 11 patients (54%), and in the lung positive images were found in 5/11 (45.4%) (Figure 1). These 6 cases with negative lung images had micrometastases, as evidenced by CT scan. In the only patient having bone metastases in which ^99m^Tc-depreotide scintigraphy was performed, the image was negative. ^18^F-FDG-PET scan was positive in 8/11 in the lung and in the neck in 5/11 (table 1).

### Response to treatment with somatostatin analogue

Five patients in whom ^99m^Tc-Depreotide scintigraphy positive images were found either in the neck and/or the lung were treated with the long acting somatostatin analog octreotide at doses of 30 mg during a period of 3–6 months in a pilot study. Response to treatment was evaluated by using lung CT scan and cervical ultrasonography imaging according to RECIST criteria. In 3 patients, no change in the size of the lesions was found. In the remaining 2 patients, there was a significant increase in the pulmonary lesions in one and in the other an important increase in the size of cervical lesions with signs of intratumoral necrosis in one of the lesions. Treatment was well tolerated in all cases, with no particular metabolic complication; although, one patient presented abdominal discomfort that disappeared after temporal decrease of the dose of octreotide. All treatments were stopped as no apparent modification of the evolution of the disease were found and in 2 patients evidence of progression seemed plausible. Due to the results of this first set of treatments, no new PDTC cases were included in the pilot study.

### Expression of ssts in well differentiated, poorly differentiated thyroid cancer tissue and normal thyroid tissue

**Figure 2:**
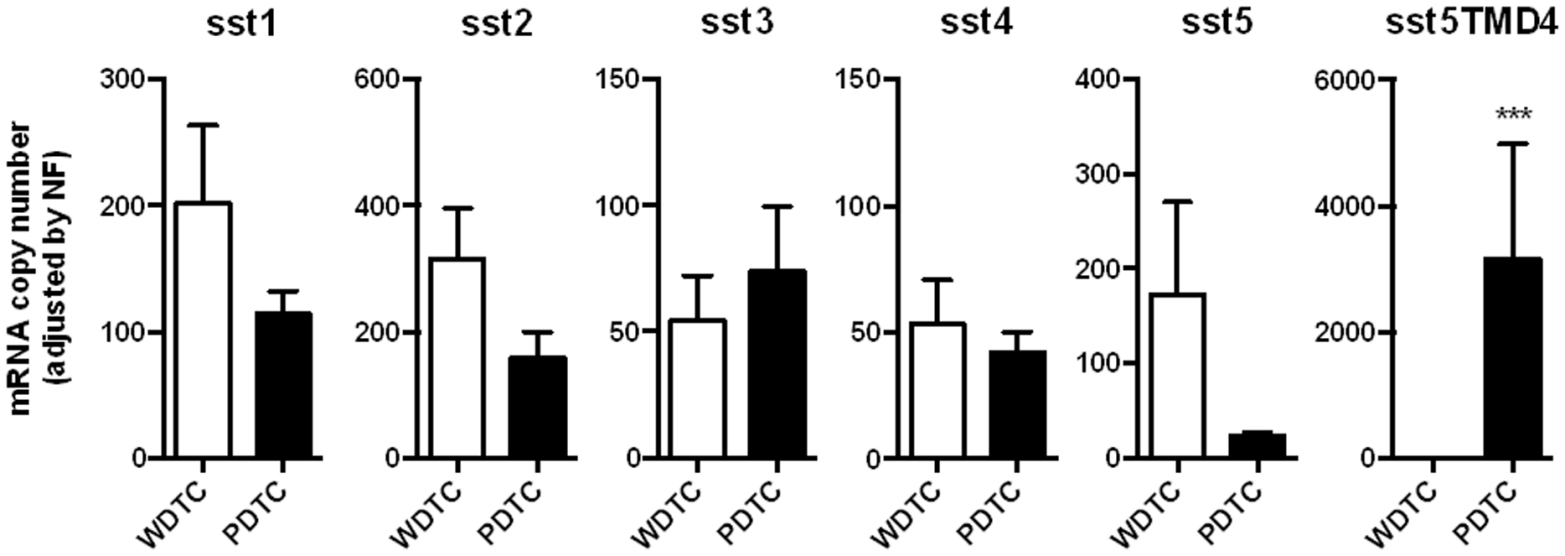
Expression profile of somatostatin receptors in thyroid cancer samples. mRNA copy number of canonical (sst1–5) and truncated (sst5TMD4) somatostatin receptors was measured by qrtRT-PCR in a battery of well-differentiated thyroid cancers (WDTC) and poorly-differentiated thyroid cancers (PDTC). Absolute mRNA level values were adjusted by a normalization factor (NF) obtained as the geometric means of the copy numbers for GAPDH and β-actin. Values represent average ± SEM (n = 19–20 patients). Asterisks (***, p<0.001) indicate values that significantly differ from WDTC.

Residual primary cancer tissue and metastases samples from the subset of patients with imaging studies and ^99m^Tc-Depreotide scintigraphy plus 9 additional patients (total = 20; Table 1) bearing PDTC were recovered for ssts expression studies. Samples from 19 patients with WDTC free of disease condition at follow-up were also included in the study and additionally samples from 7 normal thyroid glands and from a pool of normal thyroids. A variable amount of mRNA for each of the five sst-subtypes was found with no significant differences between ages and gender (data not shown) within the WDTC and PDTC groups, as well as among the normal thyroid tissue. Mean mRNA content of sst-subtypes in WDTC and PDTC is shown in Figure 2. In WDTC, sst2 was the dominant sst-subtype represented, followed by sst1>sst5>sst3 = sst4, while the expression of sst5TMD4 and sst5TMD5 were not found in WDTC samples. In the group of PDTC, the most represented sst-subtype was by far sst5TMD4, which was found to show a 23 fold-increase in expression as compared with the next most expressed sst-subtype which was sst2, followed by sst1>sst3>sst4>sst5. Expression of sst5TMD5 was not detected in PDTC. In 2 out of 20 patients (10%) with PDTC, sst5TMD4 was not detected. When comparing the sst-subtype mRNA levels between WDTC and PDTC we found that sst2 expression was higher in WDTC as compared with PDTC (317±80 *vs*. 158±42, respectively; p = 0.08). Also, expression of sst1 and sst5 tended to be increased in the PDTC group as compared with the WDTC patients (173±98 vs. 24±4 for sst1 and 202±62 vs. 114±18 for sst5; p = 0.16 and 0.12, respectively). No differences or trend to difference were found in the expression levels of sst3 or sst4 between PDTC and WDTC. Finally, all 5 patients in whom octreotide treatment was performed showed high expression levels of sst5TMD4 (mean = 2286±896).

Regarding the study performed in normal thyroid tissue, the results clearly indicate that the expression levels of sst5TMD4 is quite low in those normal samples (20±7 copies of sst5TMD4/50 ng of total RNA), which supports previous data reported by our group [18].

**Figure 3:**
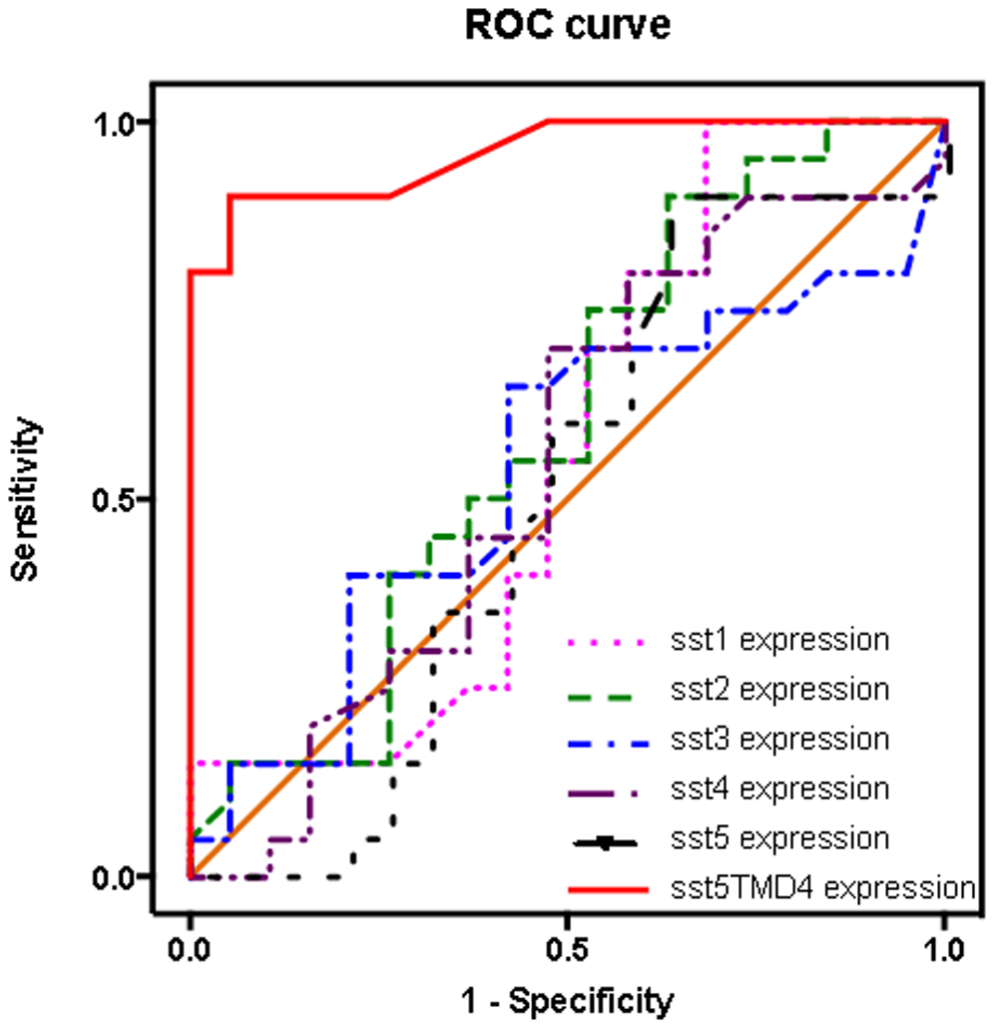
Receiver operating characteristics (ROC) curves to determine the accuracy of somatostatin receptors (sst1–5 and sst5TMD4) as diagnostic test to discriminate between well-differentiated and poorly-differentiated thyroid cancers.

ROC curves were used to estimate the performance of the different ssts expression levels to distinguish between the two diagnostic groups (patients with active disease/patients free of disease; Figure 3). The closer the ROC curve is to the upper left corner of the graphic (i.e., higher sensitivity and specificity), the higher the overall accuracy of the marker used. While expression of the canonical sst1-sst5 had a lower ability to distinguish between the two diagnostic groups (ROC curves similar to the reference line), the ROC curve of the truncated sst5TMD4 expression levels was significantly different from the reference line (area under the curve = 0.957; p<0.0001). Indeed, a cut score of 5 copies of the sst5TMD4, which coincided approximately with the mean of the expression of the patients free of disease, yielded a sensitivity of 90% and a specificity of 84%. An optimal score greater than or equal to 8.5 copies had 90% sensitivity and specificity. Therefore, our results clearly indicate that the expression of truncated sst5TMD4 variant is the only marker that could be used in a sensitive and specific manner.

## Discussion

RAI-refractory PDTC is found in about 5–10% of all follicular cell-derived thyroid cancers [1], [2]. These patients have a poor prognosis, as no efficacious treatments are available at present 24,25 and thus, the search for new treatment targets, mostly for stage III and IV cases, is currently ongoing [3], [4].

Somatostatin regulates many physiological functions in most of the organs of mammalian and non-mammalian species through its sst1–5 receptors [10]. Additionally, somatostatin and its analogues show antiproliferative actions, which make them useful as coadjuvant treatment especially for neuroendocrine tumors [7], apart from their usefulness in acromegaly [26]. In acromegaly, long-acting somatostatin analogues are associated with a decrease of about 20% in the size of adenomas after 6–12 months of treatment in one third of cases [27]. Moreover, radiolabeled peptide receptor targeted therapies are another potential option for some tumors bearing ssts [28]. The presence of ssts has been reported in normal thyroid tissue and in thyroid cancer [8]. Moreover, in thyroid carcinoma cell lines, somatostatin is able to inhibit cell proliferation even in anaplastic thyroid carcinoma cell lines [8]. Quantitative evaluation of ssts expression in thyroid cancer has shown some discrepancies, with some authors reporting the expression of sst1, 3 and 5 and others exclusively sst5 by using immunocytochemical techniques [8], [10]. Even with these discrepancies, expression of ssts in both follicular and parafollicular origin thyroid cancer tissue has been recently confirmed by other laboratories [9], [11], [12], [29]–[31].

During the last decade the presence of ssts in thyroid cancer has also been explored by thyroid scintigraphy, mostly using radiolabelled octreotide and depreotide, but also with other different compounds [32], [33], facilitating the localization of metastatic tissue not detected with RAI scan. Depreotide has a high affinity for sst2, 3 and 5, thus allowing an efficient acquisition of images in tissues expressing these receptors. In our PDTC patients, depreotide scintigraphy showed positive images in about 40–50% of them in the cervical recurrent tissue and/or the extracervical metastases, these latter mostly in the lung. Thus, we decided to perform a pilot study treating 5 PDTC depreotide positive patients having large metastases with the somatostatin analog octreotide, in an attempt to stabilize or decrease tumor growth with the result of either no improvement or even an apparently rapid growth in two of the cases. As a consequence, treatment with octreotide was withdrawn in all patients and new patients were not included. In the only previous similar pilot study published so far, Kolhfuerst *et al.* [13] found similar results and concluded that there was no indication for somatostatin analogues treatment in patients with advanced PDTC.

Later on in 2009 after these patients were studied and treated, our group [18] identified two new functional truncated variants of sst5 with 5 and 4 transmembrane domains. Importantly, using the acromegaly model, we demonstrated that the presence of those sst variants were etiologically linked to a lack of response to growth hormone inhibition as well as to the antiproliferative effects of somatostatin analogue treatment [20]. Based on these results, we hypothesized that patients with PDTC could potentially express sst5TMD4 and, as a result, they might be unresponsive to octreotide treatment. We therefore subsequently investigated in a retrospective manner the presence of such truncated variants of sst5, and demonstrated for the first time its exclusive expression in PDTC tissue as no presence was found in WDTC samples and normal thyroid tissue. This finding suggests that sst5TMD4 might appear in thyroid cancer as the dedifferentiation process progresses, although the ultimate mechanism of its anomalous expression is currently unknown. Thus, the detection of sst5TMD4 may be used as an additional marker of poor prognosis. This concept is supported by the ROC curves analyses indicating that sst5TMD4 identifies accurately PDTC, with a sensitivity of 90% and a specificity of 84%. In this regard, sst5TMD4 has also been recently found in poorly differentiated breast cancer [21] where it is also associated with a poor prognosis. Moreover, expression of sst5TMD4 in the MCF-7 breast cancer cell line increases malignancy features such as invasion and proliferation abilities. This potentiated malignant biological behavior is believed to be related to the increase in the activity of phosphorylated extracellular signal-regulated kinases 1 and 2, p-Akt levels, and cyclin D3 and Arp2/3 complex expression [34]. Moreover, sst2 expression, which is associated to a good response to somatostatin analogues, was found to be down-regulated in PDTC in comparison with WDTC. Interestingly, sst5TDM4 has been shown to interact physically with sst2, altering its signal transduction mechanisms and, as a consequence, blocking its antiproliferative mediated effects. Elimination of sst2 signaling also results in impairment of an autocrine inhibitory feedback loop, which may modify the cellular phenotype and increase cell proliferation [23], [35]. This could explain in part the apparent increase in tumor size in 2 of our patients treated with octreotide, as its binding to sst5TMD4 would have further stimulated the functional suppression of sst2 activity.

Although the design of the therapeutic part of the present study may have some limitations, mostly the absence of a control group, as it was a pilot study, the molecular findings of PDTC samples studied retrospectively seem to explain the apparent lack of response of octreotide treatment and supported the clinical decision of stopping the inclusion of more patients in the pilot study. Thus, with regard to the therapeutic implications of our findings, we believe that the conclusions of Kolhfuerst *et al*. [13] remain valid. There is sufficient clinical and now molecular information to state that advanced dedifferentiated thyroid cancer has biological features (i.e. high sst5TMD4 and low sst2) that might explain the lack of effect or even possible deleterious effects of somatostatin analogue treatment in such patients, or at least in those expressing sst5TMD4 in their tumors. As the molecular heterogeneity of thyroid tumors is more and more evident, further research on the sst5TMD4 variant may pave the way to better understand its molecular functions and potential use as a diagnostic or even therapeutic tool in thyroid cancer.
